# Travel-related schistosomiasis, strongyloidiasis, filariasis, and toxocariasis: the risk of infection and the diagnostic relevance of blood eosinophilia

**DOI:** 10.1186/1471-2334-11-84

**Published:** 2011-04-05

**Authors:** Gijs G Baaten, Gerard J Sonder, Tom van Gool, Joan A Kint, Anneke van den Hoek

**Affiliations:** 1 Department of Infectious Diseases, Public Health Service (GGD) Amsterdam, Nieuwe Achtergracht 100, PO Box 2200, 1000 CE Amsterdam, The Netherlands; 2 Department of Internal Medicine, Division of Infectious Diseases, Tropical Medicine and AIDS, Academic Medical Centre, Meibergdreef 9, 1105 AZ Amsterdam, The Netherlands; 3 National Coordination Centre for Traveler's Health Advice (LCR), Nieuwe Achtergracht 100, PO Box 1008, 1000 BA Amsterdam, The Netherlands; 4 Department of Microbiology, Parasitology Section, Academic Medical Centre, Meibergdreef 9, 1105 AZ Amsterdam, The Netherlands

**Keywords:** helminth infection, eosinophilia, travel, prospective study

## Abstract

**Background:**

This study prospectively assessed the occurrence of clinical and subclinical schistosomiasis, strongyloidiasis, filariasis, and toxocariasis, and the screening value of eosinophilia in adult short-term travelers to helminth-endemic countries.

**Methods:**

Visitors of a pre-travel health advice centre donated blood samples for serology and blood cell count before and after travel. Samples were tested for eosinophilia, and for antibodies against schistosomiasis, strongyloidiasis, filariasis, and toxocariasis. Previous infection was defined as seropositivity in pre- and post-travel samples. Recent infection was defined as a seroconversion. Symptoms of parasitic disease were recorded in a structured diary.

**Results:**

Previous infection was found in 112 of 1207 subjects: schistosomiasis in 2.7%, strongyloidiasis in 2.4%, filariasis in 3.4%, and toxocariasis in 1.8%. Recent schistosomiasis was found in 0.51% of susceptible subjects at risk, strongyloidiasis in 0.25%, filariasis in 0.09%, and toxocariasis in 0.08%. The incidence rate per 1000 person-months was 6.4, 3.2, 1.1, and 1.1, respectively. Recent infections were largely contracted in Asia. The positive predictive value of eosinophilia for diagnosis was 15% for previous infection and 0% for recent infection. None of the symptoms studied had any positive predictive value.

**Conclusion:**

The chance of infection with schistosomiasis, strongyloidiasis, filariasis, and toxocariasis during one short-term journey to an endemic area is low. However, previous stay leads to a cumulative risk of infection. Testing for eosinophilia appeared to be of no value in routine screening of asymptomatic travelers for the four helminthic infections. Findings need to be replicated in larger prospective studies.

## Background

Helminth infections are a major health problem in the developing world. Due to increased travel to developing countries and changes in travel behavior, they have gained importance in the developed world, particularly among travelers [1].

Untreated helminth infection may result in long-term adverse outcomes. Because symptomatology can be absent or non-specific, blood eosinophil count is commonly used as a screening tool [2-4]. Schistosomiasis, strongyloidiasis, filariasis, and toxocariasis are four common helminth infections associated with eosinophilia [4].

*Schistosoma *trematodes can penetrate human skin exposed to contaminated fresh water. Endemic in Africa, Central and South America, and Asia, about 200 million people worldwide are infected [5,6]. The major species give rise primarily to intestinal, hepatic and urinary pathology [7].

The nematode *Strongyloides stercoralis *can penetrate intact skin after contact with contaminated soil. It is endemic in tropical, subtropical and temperate regions, affecting 30-100 million people. Manifestations range from asymptomatic to chronic symptomatic disease [8,9].

Filarial morbidity is caused primarily by 4 nematode species. Of these, *Wuchereria bancrofti *and *Brugia malayi *are transmitted by mosquitoes and reside in the lymphatics. *W. bancrofti *is endemic throughout the developing world; *B. malayi *is limited to Asia. More than 120 million people worldwide are infected [7]. *Onchocerca volvulus *is transmitted by blackflies and resides in subcutaneous nodules and in the eye. It is endemic mainly in sub-Saharan Africa, with additional foci in Central and South America and the Middle-East. About 17.7 million people are infected [10]. *Loa loa *is transmitted by flies and causes subcutaneous swellings. It is endemic in sub-Saharan Africa [7].

Toxocariasis is caused by ingestion of eggs of the nematodes *Toxocara canis *or *Toxocara cati*, via contaminated soil. It is distributed worldwide. Manifestations vary with regard to which tissue has been invaded [11].

Research on the prevalence and incidence of these infections among travelers is scarce. Studies on the predictive value of eosinophilia for asymptomatic helminth infection have shown different correlations, and its diagnostic relevance remains controversial [3,4]. Moreover, most studies use retrospective study designs.

This study prospectively estimates the prevalence and incidence of schistosomiasis, strongyloidiasis, filariasis, and toxocariasis based on serologic testing in a cohort of short-term travelers to endemic areas. It evaluates risk factors, and assesses the diagnostic relevance of eosinophilia.

## Methods

### Study population

A prospective study was performed among persons attending the travel clinic of the Public Health Service Amsterdam from October 2006 to October 2007. All persons 18 years and older were eligible if planning to travel for 1-13 weeks to one or more developing countries. As defined by the United Nations Development Agency (UNDA), these countries were in the Caribbean and Central and South America; Western, Middle, Eastern and Southern Africa; South-Eastern Asia and China, and the South-Central Asian area of Afghanistan, Bangladesh, Bhutan, India, Nepal, Pakistan, and Sri Lanka [12].

All participants were seen by a medical doctor or nurse specialised in travel medicine. They received vaccinations, a prescription for anti-malarial chemoprophylaxis if required, and oral and written information about how to avoid acquiring travel-related diseases, based on Dutch National Guidelines on Traveller's Health Advice.

### Survey methods

Before departure and 2 to 6 weeks after return, participants donated venous blood samples for serology and blood cell count. Before departure, a standard questionnaire was used to collect data on socio-demographics, including purpose of travel. Participants were also asked to keep a structured travel diary, recording symptoms and signs of parasitic disease (fever, myalgia, skin infection, and gastro-intestinal disorder), from the day they arrived at the destination until 2 weeks after return, to encompass incubation periods of acute travel-related infections. Thus 'travel-related' refers to the period of travel and the two weeks thereafter.

Destinations were grouped into regions using the UNDA classification [12]. Travel duration was recorded as the total days spent in areas meeting the inclusion criteria.

The study protocol was approved by the Medical Ethics Committee of the Academic Medical Centre Amsterdam. Participants were included only after informed and written consent.

### Laboratory methods

All blood samples were immediately stored at 6°C. The total leukocyte count and the eosinophil count of both pre-travel and post-travel samples were determined within 24 hours by automated analyzer (Sysmex SE- 9000, Toa Medical Instruments, Kobe, Japan). Blood samples for serologic testing were centrifuged and frozen at -80°C within 24 hours, to be tested after all participants had returned. For serodiagnosis of *Schistosoma mansoni, haematobium*, and *japonicum*, an indirect hemagglutination assay (IHA) with adult *S. mansoni *worm antigens (Fumouze Laboratories, Levallois-Perret, France) and an enzyme-linked immunosorbent assay (ELISA) with *S. mansoni *soluble egg antigens were used [13]. For *S. stercoralis*, an in-house ELISA based on antigen of *S. stercoralis *was used [14]. For filariasis, a commercially available ELISA on microtitration wells sensitized with *Acanthocheilonema viteae *somatic antigens was used (Bordier Affinity Products, Crissier, Switzerland). For *toxocariasis*, a commercially available ELISA on microtitration wells sensitized with *T. canis *E/S larval antigens was used (Bordier Affinity Products, Crissier, Switzerland).

Sensitivity and specificity in clinical settings were 100% and 93% for the combined IHA and ELISA for *Schistosomiasis*; 93% and 95% for the ELISA for *Strongyloides*; 95% and 98% for the ELISA for filariasis; and 91% and 86% for the ELISA for *Toxocariasis *[13-16].

For participants whose post-travel sample tested positive, pre-travel samples were also tested. Previous infection was defined as a seropositive pre- and post-travel sample. Recent infection was defined as a seropositive post-travel sample with a seronegative pre-travel sample for a given disease, i.e. seroconversion. In a traveler whose post-travel sample was just above the cut-off and whose pre-travel sample was just below the cut-off, previous infection was assumed.

### Data analysis

Participants were considered to be at risk for a specific infection if they had spent one day or more in an endemic area. Endemicity was based on information from The Global Infectious Diseases and Epidemiology Online Network [17]. Participants were considered susceptible for a given disease in case of a seronegative pre-travel sample.

Attack rates per 100 travelers were calculated by dividing the number of seroconversions by the total number of participants at risk and susceptible for the given infection. Incidences rates per 1000 person-months were calculated by dividing the number of seroconversions by the total number of months in which participants were at risk for infection. Attack rates and incidence rates were calculated using OpenEpi, version 2.3 [18].

Eosinophilia was defined as an eosinophil count of more than 450 per mm^3^. The predictive value of eosinophilia defined as ≥8% or ≥10% eosinophils per total leukocyte count was also examined. For travelers with recent infection, the eosinophil counts of post-travel samples were analyzed. For travelers with previous helminth infection and for seronegative travelers, the pre-travel eosinophil counts were analyzed. The sensitivity of eosinophilia for the serology-based presence of parasitic infection was calculated by dividing the number of seropositive participants with eosinophilia by the number of all seropositive participants. Specificity was calculated by dividing the number of seronegative participants without eosinophilia by the number of all seronegative participants. The positive predictive value (PPV) was calculated by dividing the number of seropositive participants with eosinophilia by the number of all participants with eosinophilia. The negative predictive value (NPV) was calculated by dividing the number of seronegative participants without eosinophilia by the number of all participants without eosinophilia.

To calculate risk factors for previous infection, SPSS for Windows version 17.0 (SPSS Inc., Chicago) was used to obtain prevalence rates (PRs), univariate and multivariate prevalence rate ratios (PRRs), and 95% confidence intervals (CIs), by means of logistic regression modeling. All variables with an overall p-value < 0.05 in univariate analysis were included in multivariate analysis. Statistical interactions between variables were checked for all variables. If significant (p < 0.05), they were included in multivariate analysis.

Chi-square tests were used to evaluate the predictive value of travel-related symptoms for recent infection. Fever was defined as a body temperature ≥38°C. A p-value < 0.05 was considered statistically significant.

## Results

### Study population

The study began with 1276 subjects who intended to travel to the developing world. Of these, 69 (5.4%) were excluded: 33 were lost to follow-up, 23 had their travel arrangements cancelled, 7 did not supply the post-travel blood donation, 3 changed travel plans such that inclusion criteria were not met, 2 did not complete the structured diary, and for 1 case, the post-travel sample was lost.

Table 1 describes the remaining 1207 subjects. The vast majority were native Dutch tourists travelling for holiday purposes. On this trip, all visited one or more countries endemic for *S. stercoralis*, filariasis, and toxocariasis, and 1006 visited one or more countries endemic for schistosomiasis. The characteristics of the latter did not differ from the characteristics of all 1207 subjects (data not shown).

**Table 1:** Characteristics of a cohort of 1207 travelers to developing countries, attending a Dutch Travel Health Clinic for pre-travel health advice, October 2006 - October 2007

Number of subjects	1207	
Male sex	521	43%
		
Median age in years °	38	(29-51)
		
Country of birth		
Netherlands	1049	87%
Other Western country	69	6%
Non-Western country	89	7%
		
Current travel destination		
South-east Asia	375	31%
South and West Asia	147	12%
East Asia	98	8%
		
South America	219	18%
Central America and Caribbean	135	11%
		
East Africa	140	12%
West and Middle Africa	113	9%
Southern Africa	67	6%
		
Primary purpose of travel		
Tourism	1032	86%
Work or education	99	8%
Visiting friends and/or relatives	76	6%
		
Median travel duration in days °	21	(16-28)
		
Median interval between return from		
travel and blood donation, in days °	25	(21-29)
		
Previous travel to a developing country		
Never	221	18%
1 to 5 times	703	58%
6 times or more	283	23%
		
Previous travel destinations		
Asia	670	56%
Latin America	546	45%
Africa	528	44%

° Interquartile range between brackets		

### Serology suggestive for previous and recent infection

Serology suggestive for previous infection was found 124 times in 112 of 1207 subjects: for *schistosoma spp*. in 32 (2.7%; 95%CI: 1.9-3.7%); for *S. stercoralis *in 29 (2.4%; 95%CI: 1.6-3.4%); for filariasis in 41 (3.4%; 95%CI: 2.5-4.5%); and for *toxocara spp*. in 22 (1.8%; 95%CI: 1.2-2.7%). Ten of 112 travelers had serology suggestive for 2 previous infections and 1 for 3 previous infections. Three of 32 (9.4%) with previous schistosomiasis and 4 of 41 (9.8%) with previous filariasis did not report birth or previous travel to a developing country.

Serology suggestive for recent infection was found in 10 cases: for schistosoma spp. in 5 of 979 susceptible subjects at risk (0.51%), for *S. stercoralis *in 3 of 1178 (0.25%), for filarial spp. in 1 of 1166 (0.086%), and for *toxocara spp*. in 1 of 1185 (0.084%). The incidence rate per 1000 person-months was 6.4 for schistosomiasis, 3.2 for strongyloidiasis, 1.1 for filariasis, and 1.1 for toxocariasis. Table 2 shows the attack rates and incidence rates, overall and per region. The characteristics, travel-related symptoms, and pre- and post-travel eosinophil counts of recently infected subjects are shown in Table 3. None had serology suggestive for more than one recent infection. The median interval between return from travel and blood donation for these ten travelers was 25 days (range 23-28).

**Table 2:** Attack rates and incidence rates of seroconversions in antibody levels schistosoma spp

Parasite	Region	No. of sero- conversions	SusceptiblesM at risk	Person-months of travel	Attack rate °		Incidence rate per 1000 person-months °	
Schistosoma spp.	All regions	5	979	782.5	0.51%	(0.19-1.1%)	6.4	(2.3-14.2)
	Africa	0	290	218.7	0%	NA	0	NA
	Asia	4	532	444.1	0.75%	(0.24-1.8%)	9.0	(2.9-21.7)
	Latin America	1	157	119.8	0.64%	(0.032-3.1%)	8.3	(0.42-41.2)
								
S. stercoralis	All regions	3	1178	936.4	0.25%	(0.065-0.69%)	3.2	(0.81-8.7)
	Africa	0	290	216.4	0%	NA	0	NA
	Asia	3	554	463.5	0.54%	(0.14-1.5%)	6.5	(1.6-17.6)
	Latin America	0	334	256.4	0%	NA	0	NA
								
Filaria spp.	All regions	1	1166	930.1	0.086%	(0.004-0.42%)	1.1	(0.054-5.3)
	Africa	0	285	214.7	0%	NA	0	NA
	Asia	1	549	459.8	0.18%	(0.009-0.90%)	2.2	(0.11-10.7)
	Latin America	0	332	255.6	0%	NA	0	NA
								
T. canis	All regions	1	1185	943.3	0.084%	(0.004-0.42%)	1.1	(0.053-5.2)
	Africa	1	293	220.9	0.34%	(0.017-1.7%)	4.5	(0.23-22.3)
	Asia	0	560	466.6	0%	NA	0	NA
	Latin America	0	332	255,8	0%	NA	0	NA

° 95% confidence interval between brackets				NA: Not applicable				

**Table 3:** Characteristics, symptoms, and eosinophil counts of subjects with serological evidence for recent infection with schistosoma spp

Serological conversion for		Sex	Age in years	Country of birth	Destination	Travel duration in days	Previous travel to a developing country	Travel-related symptoms	Eosinophil count per mm^3 ^(proportion of leukocytes)		
									Pre-travel	Post-travel	*
1	Schist	M	29	Netherlands	India	42	2 - 5×	Four weeks of watery diarrhoea	40 (1.1%)	60 (1.7%)	↑
2	Schist	M	36	Iran	Vietnam	22	1 x	None	100 (2.2%)	160 (3.1%)	↑
3	Schist	M	35	Belgium	Myanmar, Thailand, Malaysia	29	1 x	Two days of watery diarrhoea with fever	130 (2.5%)	130 (2.7%)	=
4	Schist	F	26	Netherlands	Thailand	21	0×	None	560 (7.9%)	330 (5.1%)	↓
5	Schist	F	22	Netherlands	Dominican Republic	29	0 x	Three days of watery diarrhoea	170 (3.2%)	220 (4.2%)	↑
											
6	Strong	F	59	Netherlands	China	14	2 - 5×	None	80 (1.4%)	170 (2.8%)	↑
7	Strong	F	48	Surinam	India	24	>10×	Nine days of bloody diarrhoea and fever	130 (1.9%)	100 (1.1%)	↓
8	Strong	F	65	Netherlands	India	14	6 - 10×	Two days of diarrhoea	320 (4.9%)	120 (1.4%)	↓
											
9	Fil	F	54	Netherlands	India	14	2 - 5×	Three 2-day episodes of watery diarrhoea.	80 (1.5%)	110 (2.3%)	↑
											
10	Toxo	M	53	Surinam	Angola	59	>10×	Two weeks myalgia, arthritis with skin rash	110 (2.1%)	130 (2.7%)	↑

Schis: Schistosoma spp. Strong: Strongyloides stercoralis Fil: Filaria spp. Toxo: Toxocara canis M: male F: female

*) The change in the absolute eosinophil count per mm^3^, post-travel versus pre-travel: ↑ indicating an increase, ↓ a decrease, and = no change.

### Eosinophilia

The median pre-travel eosinophil count among all 1207 subjects was 150 per mm^3 ^(95%CI: 30-560), and 55 (4.6%) had pre-travel eosinophilia. The latter did not significantly differ from subjects without eosinophilia in sociodemographics, travel characteristics, or travel-related symptoms (p>0.05).

Among the 112 subjects with previous infection, the median eosinophil count was 150 per mm^3 ^(95%CI: 30-664), and 8 (7.1%) had eosinophilia. The sensitivity of eosinophilia for serological diagnosis of previous infection was 7%, the specificity 96%, the PPV 15%, and the NPV 91%. When eosinophilia was defined as ≥8% eosinophils per total leukocyte count, the PPV was 14%. When eosinophilia was defined as ≥10% eosinophils per total leukocyte count, the PPV increased to 20%, whereas the NPV remained 91%.

Among the 10 subjects with recent infection, the median eosinophil count was 130 per mm^3 ^(95%CI: 60-330); none had eosinophilia. Thus, the sensitivity and PPV of eosinophilia for the serological diagnosis of recent infection were 0%; the specificity and NPV were 95% and 99%, respectively. When eosinophilia was defined as ≥8% or ≥10% eosinophils per total leukocyte count, the PPV remained 0%.

### Risk factors and markers for previous and recent infection

Table 4 shows the PRs and PRRs with accompanying 95% confidence intervals and p-values for potential risk factors for previous infection. The prevalence rate was significantly higher for male travelers, for travelers with frequent previous travel to a developing country, and for travelers going for work/education or visiting friends and/or relatives. There were no interactions indicating effect-modification.

**Table 4:** Potential risk factors for previous infection with schistosoma spp

	Total	Antibody-positive		Univariate analysis			Multivariate analysis		
		N	PR	PRR	(95%CI)	p-value	PRR	(95%CI)	p-value
Number of subjects	1207	112	9.3%	NA					
									
Sex									
Male	521	60	11.5%	1.6	(1.1-2.3)	**0.020**	1.6	(1.1-2.4)	**0.020**
Female	686	52	7.6%	1			1		
									
Median age in years	38	43		1.01	(0.99-1.03)	0.076			
(Interquartile range)	(29-51)	(31-54)							
									
Country of birth									
Western country	1118	100	8.9%	1			1		
Non-Western country	89	12	13.5%	1.6	(0.84-3.0)	0.16	1.1	(0.58-2.2)	0.71
									
Previous travel to a developing country						**0.022**			0.14
Never	221	12	5.4%	1			1		
1 to 5 times	703	64	9.1%	1.7	(0.92-3.3)	0.086	1.7	(0.83-3.3)	0.15
6 times or more	283	36	12.7%	2.5	(1.3-5.0)	**0.007**	2.2	(1.01-4.7)	**0.048**
									
Previous travel destinations									
Not Asia	537	41	7.6%	1					
Asia	670	71	10.6%	1.4	(0.96-2.1)	0.08			
									
Not Latin America	661	51	7.7%	1			1		
Latin America	546	61	11.2%	1.5	(1.02-2.2)	**0.040**	1.2	(0.78-1.9)	0.41
									
Not Africa	679	56	8.2%	1					
Africa	528	56	10.6%	1.3	(0.89-1.9)	0.16			
									
Primary purpose of current travel						**0.005**			**0.010**
Tourism	1032	84	8.1%	1			1		
Work or education	99	15	15.2%	2.0	(1.1-3.6)	**0.021**	1.9	(1.1-3.6)	**0.030**
Visiting friends/relatives	76	13	17.1%	2.1	(1.2-4.4)	**0.009**	2.2	(1.2-4.3)	**0.016**

N: number of cases									
PR: prevalence rate									
PRR: prevalence rate ratio									
CI: confidence intervals									
NA: not applicable									

Previous infection was not related to disease symptoms during the current trip or eosinophilia: ORs equaled 1.0 with p-values > 0.05 (not shown in Table).

Compared to all seronegative subjects, the 10 subjects with recent infection were more often born in a non-western country (30% vs. 7%; OR 5.8, 95%CI: 1.2-22.7; p-value: 0.031), and their current travel destination was more often Asia (83% vs. 47%; OR 5.6, 95%CI: 1.4-38.0; p-value: 0.014). Recent infection was not related to travel duration, the interval between return from travel and blood donation, disease symptoms nor to eosinophilia (ORs equaled 1.0 with p-values > 0.05).

## Discussion

In this prospective study the serology-based attack rates and incidence rates of recent schistosomiasis, strongyloidiasis, filariasis, and toxocariasis in short-term travelers to endemic areas were low. Recent infection with any of the 4 parasites was found in only 0.8% of travelers, and disease-specific incidences ranged between 1.1 and 6.4 per 1000 person-months of travel. However, as much as 9.3% of travelers had previous infection, indicating that exposure from a previous stay raises risk of infection. Indeed, previous infection was related to a history of frequent travel to developing countries. Since toxocariasis is endemic in developed and developing countries, rates of previous toxocara infections are of limited value from a travel-medicine perspective.

None of the diary-recorded symptoms had any predictive value for seroconversion or pre-existing seropositivity. The PPV of eosinophilia was low, being 15% for diagnosis of previous infection and 0% for recent infection.

Studies on travel-related helminth infection have reported schistosomiasis in 1.3-1.6% of travelers, strongyloidiasis in 0.1-1.2%, and filariasis in 0.6-1.0% [3,19-21]. A study on travel-related toxocariasis could not be identified. Eosinophilia was found in only 38-65% of patients with filariasis, strongyloidiasis, and/or schistomiasis, and its PPV was low [1,3,4]. All aforementioned studies used a retrospective and cross-sectional study design based on immigrants, expatriates, or tourists who sought medical attention after return. They are influenced by referral bias, cannot compare characteristics to those who have remained well, and lack valid denominator data to determine absolute risk. Thus comparing their findings with ours is difficult. Nevertheless, as the PPV is generally proportional to disease occurrence, the PPV of eosinophilia was expected to be even lower in our cohort of asymptomatic travelers with a low incidence of infection. Also, eosinophilia can arise from other medical conditions, including allergic disorders. One can conclude that routine screening for eosinophilia of asymptomatic travelers after return apparently has no value.

In our study, most of the recent infections were contracted in India and South-east Asia. In other studies, most parasitic infections, in particular schistosomiasis and filariasis, were contracted in sub-Saharan Africa [6,20-24]. These differences may be explained by differences in study population and risk behavior. In the retrospective studies the decision to perform a diagnostic test was based on the physicians' expectations about disease endemicity: if schistosomiasis is not expected in a traveler from India, one doesn't test for it. Although the seroconversion might be false-positive, cases of *S. haematobium *in India have been reported in the past [17], and the helminth might still be present today. In our prospective study the risk of infection was subject to the travelers' expectations about endemicity and their own risk behavior. Thus travelers to Africa may have been more cautious than those to Asia. More studies on disease endemicity and risk behavior are needed.

We also found that positive serology was related to male gender and non-touristic travelling, but not to symptomatology, as reported elsewhere [7,9,11,19-22].

The best methodological approach for estimating incidence rates of clinical and subclinical travel-related helminth infections is to follow a cohort of travelers prospectively, as we did [25]. Only 5.4% of participants were lost to follow-up, which strengthens our findings. Nevertheless, our study has some limitations.

First, given the low number of seroconversions, our sample size is too small to give a precise estimate of disease incidence.

Second, there may have been selection bias. Although our subjects are comparable to the average traveler, they were all seeking pre-travel health advice [26]. Thus they perhaps had a more than average health awareness, particularly after receiving oral and written travel advice, learning about the study, and agreeing to participate. However, the effect of pre-travel consultation tends to wane over time, and travelers may seek consultation because they plan to visit more risky areas [21,27]. Also, we did not have information on risk behavior at the destination.

Third, the 112 previously infected travelers may have been re-infected with the same parasite during this study. Indeed, previously infected travelers may have more risky behavior. As serologic tests cannot discriminate a new infection from a re-infection in these travelers, we may have underestimated attack and incidence rates. However, in all persons with evidence for previous infection, the post-travel antibody level equaled the pre-travel antibody level.

Finally, serologic testing has additional drawbacks. A seroconversion with a slow rise in antibody levels could have been missed, leading to underestimation. However, most subjects donated blood more than 21 days after return (Table 1), enough to detect a rise in antibodies in most cases. The tests for strongyloides and filariasis can cross-react mutually and with echinococcus spp. and other nematodes, causing overestimation [19,28]. When attack rates are very low, false positive results can occur just by chance. This may explain why 7 subjects tested positive for schistosomiasis or filariasis without reporting birth or travel in an endemic country, although under-reporting of travel-history cannot be excluded. Blood, stool and urine microscopy could have yielded valuable additional information, but were not part of the study protocol for logistical reasons [2]. Nevertheless, without a true gold standard for ruling out parasitic infections, true sensitivity and specificity measurements are difficult to obtain, as are valid prevalence and incidence rates.

## Conclusions

Attack rates and incidence rates of schistosomiasis, strongyloidiasis, filariasis, and toxocariasis during one short-term journey to an endemic area are low; routine serological testing of returned travelers appears to be of no value. However, previous stay or travel lead to a cumulative risk of infection. Infection rates are difficult to assess, as they depend on geographic distribution of helminths, travel behavior and pre-existing immunity of travelers, and the characteristics of diagnostic assays. Determining the blood eosinophil count appeared to be of no value for routine screening for helminth infections in asymptomatic travelers, because of its very poor PPV. To improve preventive education and post travel follow-up strategies, more prospective studies on risk and risk behavior are needed.

## Competing interests

The authors declare that they have no competing interests.

## Authors' contributions

GB designed and conducted the study, analysed the data and wrote the article. GS designed the study and contributed to the article. TvG performed the laboratory analyses and contributed to the article. JK collected and analysed data. JARvdH designed the study, contributed to the article, and was guarantor.

All authors read and approved the final manuscript.

## Pre-publication history

The pre-publication history for this paper can be accessed here:

http://www.biomedcentral.com/1471-2334/11/84/prepub

## References

[B1] Bierman WF Wetsteyn JC van Gool T Presentation and diagnosis of imported schistosomiasis: relevance of eosinophilia, microscopy for ova, and serology J Travel Med 2005 12 9 13 10.2310/7060.2005.00003 15996461

[B2] Checkley AM Chiodini PL Dockrell DH Bates I Thwaites GE Booth HL Brown M Wright SG Grant AD Mabey DC Whitty CJM Sanderson F On behalf of the British Infection Society and The Hospital for Tropical Diseases Eosinophilia in returning travellers and migrants from the tropics: UK recommendations for investigation and initial management J Infection 2010 60 1 20 10.1016/j.jinf.2009.11.003 19931558

[B3] Libman MD MacLean JD Gyorkos TW Screening for schistosomiasis, filariasis, and strongyloidiasis among expatriates returning from the tropics Clin Infect Dis 1993 17 353 9 10.1093/clinids/17.3.353 8218675

[B4] Schulte C Krebs B Jelinek T Nothdurft HD von Sonnenburg F Löscher T Diagnostic significance of blood eosinophilia in returning travelers Clin Infect Dis 2002 34 407 11 10.1086/338026 11753824

[B5] World Health Organisation Expert Committee on the Control of Schistosomiasis Prevention and control of schistosomiasis and soil-transmitted helminthiasis: report of a WHO expert committee 2002 Geneva, Switzerland WHO technical report series 912 12592987

[B6] Chitsulo L Engels D Montresor A Savioli L The global status of schistosomiasis and its control Acta Tropica 2000 77 41 51 10.1016/S0001-706X(00)00122-4 10996119 PMC5633072

[B7] Heymann DL Control of Communicable Diseases Manual 2005 American Public Health Association Washington DC, United States of America

[B8] Lim S Katz K Krajden S Fuksa M Keystone JS Kain KC Complicated and fatal Strongyloides infection in Canadians: risk factors, diagnosis and management CMAJ 2004 171 479 84 10.1503/cmaj.1031698 15337730 PMC514646

[B9] Olsen A van Lieshout L Marti H Polderman T Polman K Steinmann P Stothard R Thybo S Verweij JJ Magnussen P Strongyloidiasis--the most neglected of the neglected tropical diseases? Trans R Soc Trop Med Hyg 2009 103 967 72 10.1016/j.trstmh.2009.02.013 19328508

[B10] Udall DN Recent updates on onchocerciasis: diagnosis and treatment Clin Infect Dis 2007 44 53 60 10.1086/509325 17143815

[B11] Despommier D Toxocariasis: clinical aspects, epidemiology, medical ecology, and molecular aspects Clin Microbiol Rev 2003 16 265 72 10.1128/CMR.16.2.265-272.2003 12692098 PMC153144

[B12] United Nations Economic and Social Development Agency Definition of major areas and regions http://esa.un.org/unpp/definition.html (Accessed: August 6, 2009)

[B13] Van Gool T Vetter H Vervoort T Doenhoff MJ Wetsteyn J Overbosch D Serodiagnosis of imported schistosomiasis by a combination of a commercial indirect hemagglutination test with Schistosoma mansoni adult worm antigens and an enzyme-linked immunosorbent assay with S. mansoni egg antigens J Clin Microbiol 2002 40 3432 7 10.1128/JCM.40.9.3432-3437.2002 12202589 PMC130766

[B14] Van Doorn HR Koelewijn R Hofwegen H Gillis H Wetsteyn JC Wismans PJ Sarfati C Vervoort T van Gool T Use of enzyme-linked immunosorbent assay and dipstick assay for detection of Strongyloides stercoralis infection in humans J Clin Microbiol 2007 45 438 42 10.1128/JCM.01735-06 17151215 PMC1829047

[B15] Houze S Eisele L Vaslin L Le Bras J Evaluation d'un réactif Elisa pour la sérologie de la filariose. Société Française de Parasitologie 2003 Marseille, France

[B16] Jacquier P Gottstein B Stingelin Y Eckert J Immunodiagnosis of toxocarosis in humans: evaluation of a new enzyme-linked immunosorbent assay kit J Clin Microbiol 1991 29 1831 5 1774303 10.1128/jcm.29.9.1831-1835.1991 PMC270219

[B17] GIDEON Informatics The Global Infectious Diseases and Epidemiology Online Network 1994 http://web.gideononline.com/web/epidemiology/(Accessed: June 29, 2009)

[B18] Dean AG Sullivan KM Soe MM OpenEpi: Open Source Epidemiologic Statistics for Public Health 2009 http://www.OpenEpi.com Version 2.3. (Accessed: 6 August 2009)

[B19] Boggild AK Yohanna S Keystone JS Kain KC Prospective analysis of parasitic infections in Canadian travelers and immigrants J Travel Med 2006 13 138 44 10.1111/j.1708-8305.2006.00032.x 16706944

[B20] Lipner EM Law MA Barnett E GeoSentinel Surveillance Network: Filariasis in travellers presenting to the GeoSentinel Surveillance Network PLoS Negl Trop Dis 2007 1 e88 10.1371/journal.pntd.0000088 18160987 PMC2154385

[B21] Nicolls DJ Weld LH Schwartz E Reed C von Sonnenburg F Freedman DO Kozarsky PE GeoSentinel Surveillance Network: Characteristics of schistosomiasis in travelers reported to the GeoSentinel Surveillance Network 1997-2008 Am J Trop Med Hyg 2008 79 729 34 18981513

[B22] Grobusch MP Mühlberger N Jelinek T Bisoffi Z Corachan M Harms G Matteelli A Fry G Hatz C Gjorup I Schmid ML Knobloch J Puente S Bronner U Kapaun A Clerinx J Nielsen LN Fleischer K Beran J da Cuncha S Schulze M Myrvang B Hellgren U Imported schistosomiasis in Europe: sentinel surveillance data from TropNetEurop J Travel Med 2003 10 164 9 10.2310/7060.2003.35759 12757691

[B23] TropNetEurop Schistosomiasis in 2008 TropNetEurop Friends & Observers Sentinel Surveillance Report 2009

[B24] Whetham J Day JN Armstrong M Chiodini PL Whitty CJ Investigation of tropical eosinophilia; assessing a strategy based on geographical area J Infect 2003 46 180 185 10.1053/jinf.2002.1108 12643868

[B25] Leder K Wilson ME Freedman DO Torresi J A comparative analysis of methodological approaches used for estimating risk in travel medicine J Travel Med 2008 15 263 72 10.1111/j.1708-8305.2008.00218.x 18666927

[B26] Van Herck K Castelli F Zuckerman J Nothdurft H Van Damme P Dahlgren AL Gargalianos P Lopez-Velez R Overbosch D Caumes E Walker E Gisler S Steffen R Knowledge, attitudes and practices in travel-related infectious diseases: the European airport survey J Travel Med 2004 11 3 8 10.2310/7060.2004.13609 14769280

[B27] Whitty CJ Carroll B Armstrong M Dow C Snashall D Marshall T Chiodini PL Utility of history, examination and laboratory tests in screening those returning to Europe from the tropics for parasitic infection Trop Med Int Health 2000 5 818 23 10.1046/j.1365-3156.2000.00642.x 11123831

[B28] Sudarshi S Stümpfle R Armstrong M Ellman T Parton S Krishnan P Chiodini PL Whitty CJM Clinical presentation and diagnostic sensitivity of laboratory tests for Strongyloides stercoralis in travellers compared with immigrants in a non-endemic country Trop Med Int Health 2003 8 728 32 10.1046/j.1365-3156.2003.01069.x 12869094

